# Supplementary material for: An unusual early-diverging plesiosauroid from the Lower Jurassic Posidonia Shale of Holzmaden, Germany
Source: PeerJ. 2025 Aug 4;13:e19665. doi: 10.7717/peerj.19665 (PMC12330822; doi:10.7717/peerj.19665)

**Supplementary material 3 for:**

An unusual early-diverging plesiosauroid from the Lower Jurassic Posidonia Shale of Holzmaden, Germany

Sven Sachs and Daniel Madzia

**Figure S1.** Parsimony analysis using equal weights. Strict consensus tree. Numbers on nodes show Bremer support values.


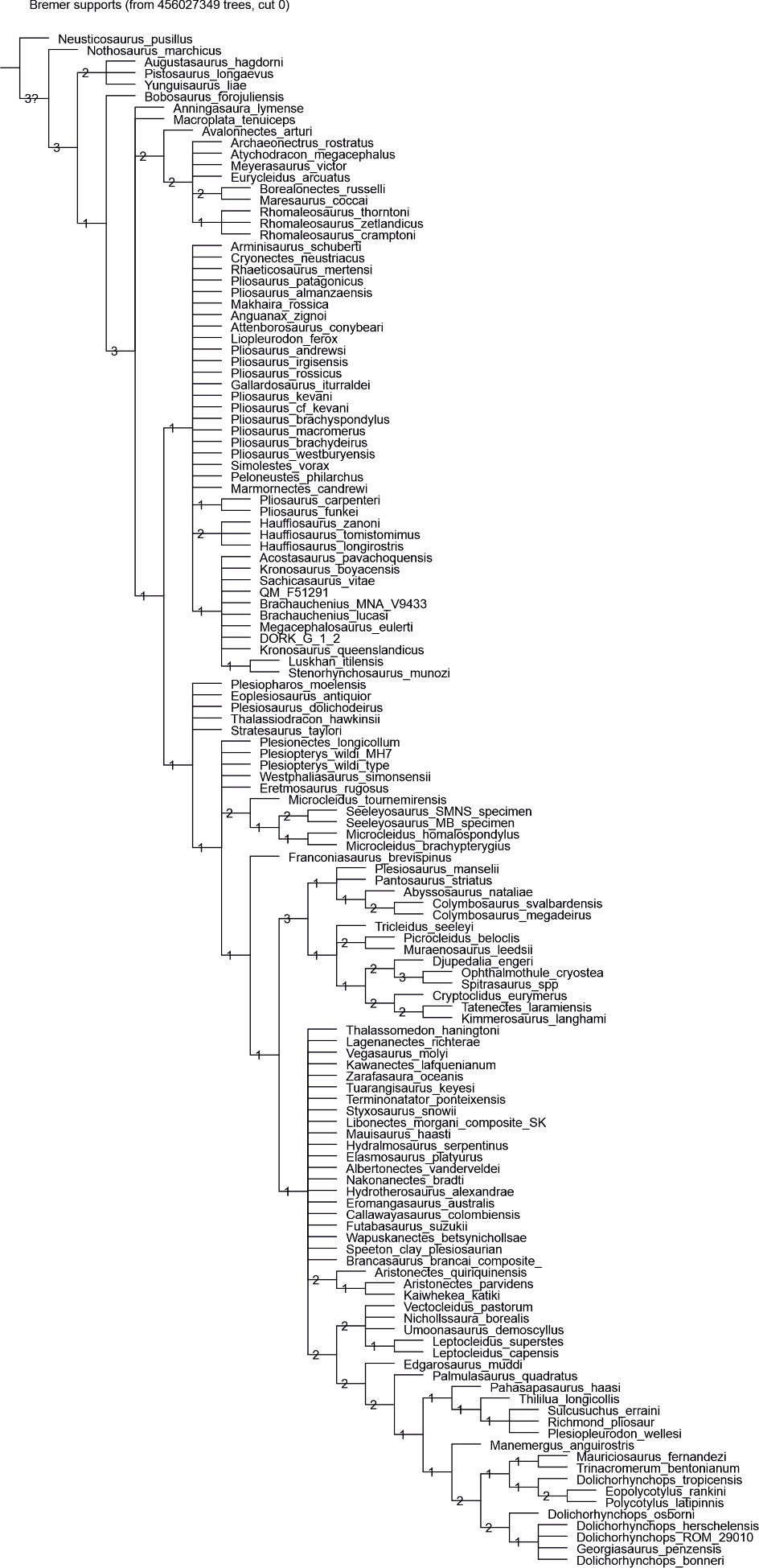


**Figure S2.** Parsimony analysis using equal weights. Majority rule consensus tree. Numbers on nodes show the percentage of the most parsimonious trees that found the nodes.


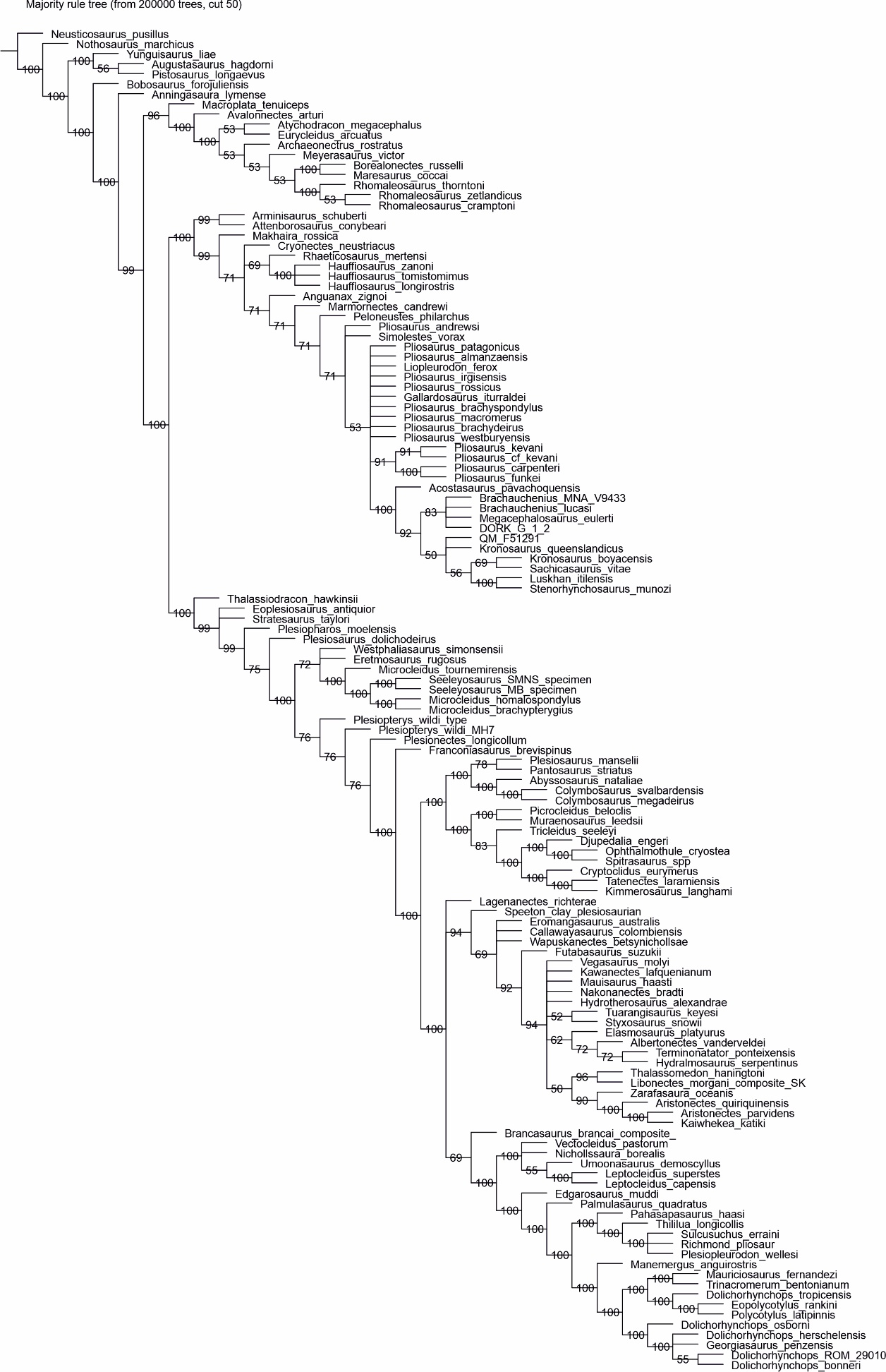


**Figure S3.** Parsimony analysis with implied weighting (*K* = 9). Strict consensus tree.


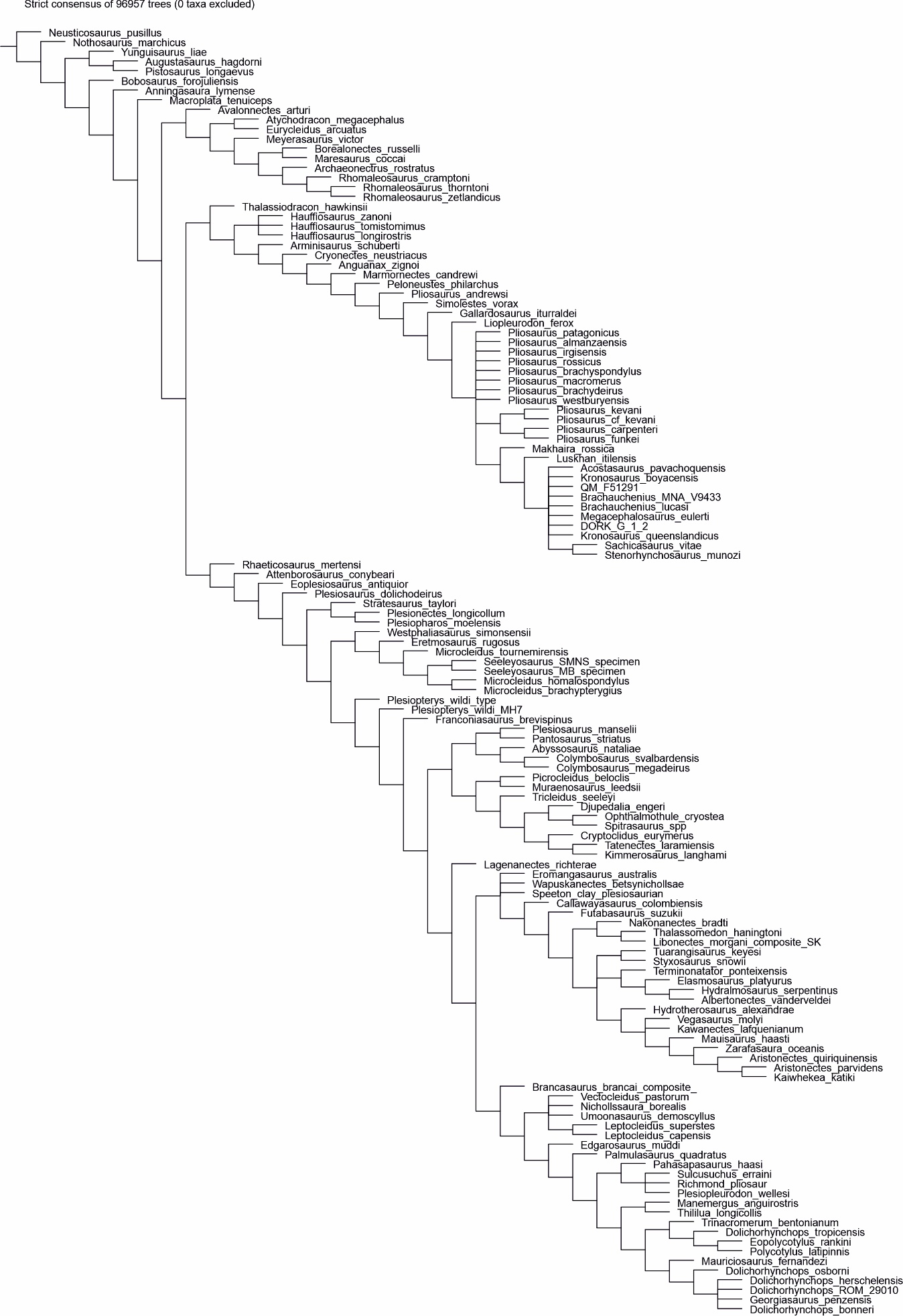


**Figure S4.** Parsimony analysis with implied weighting (*K* = 9). Symmetric Resampling.


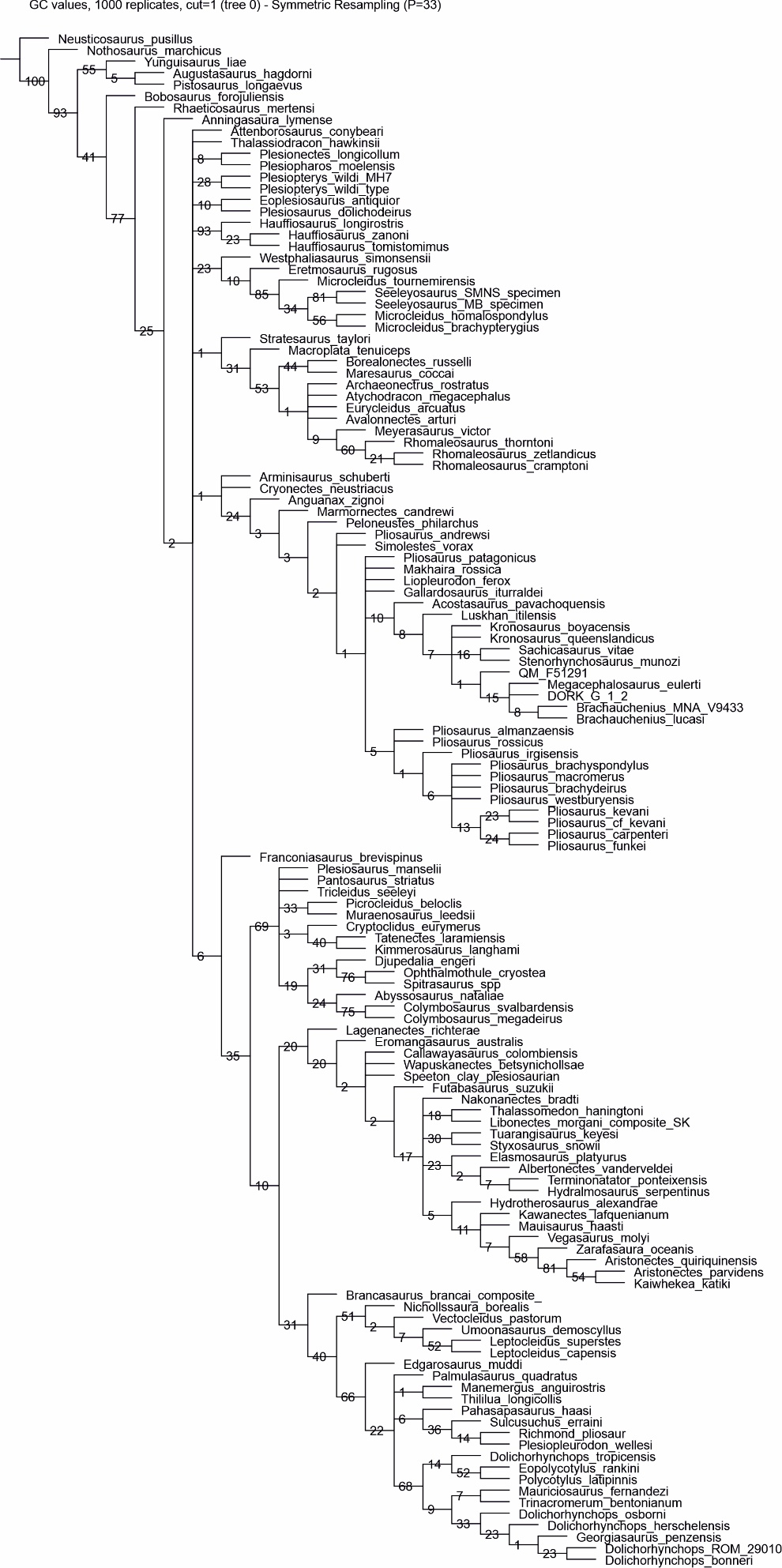


**Figure S5.** Parsimony analysis with implied weighting (*K* = 12). Strict consensus tree.


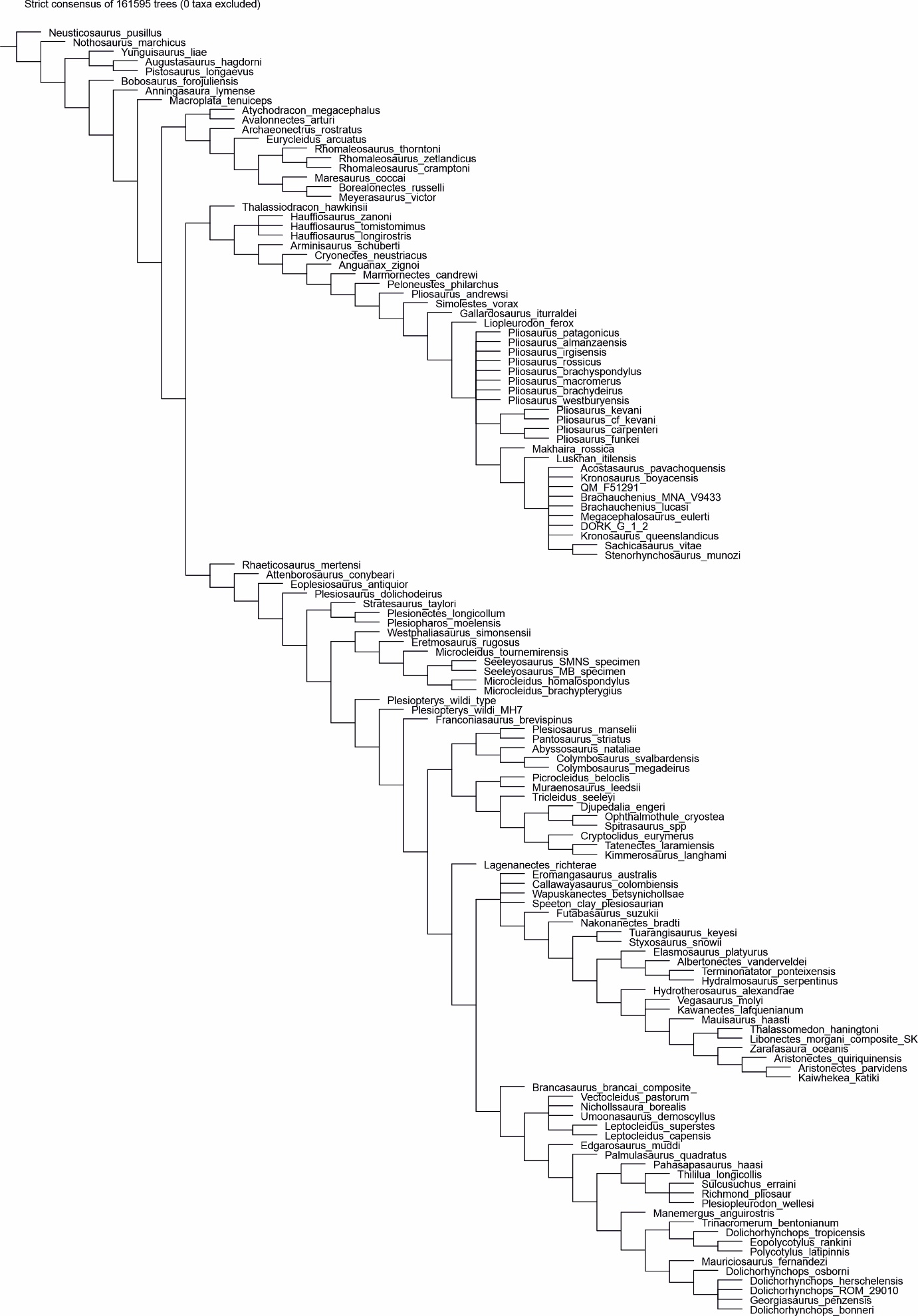


**Figure S6.** Parsimony analysis with implied weighting (*K* = 12). Symmetric Resampling.


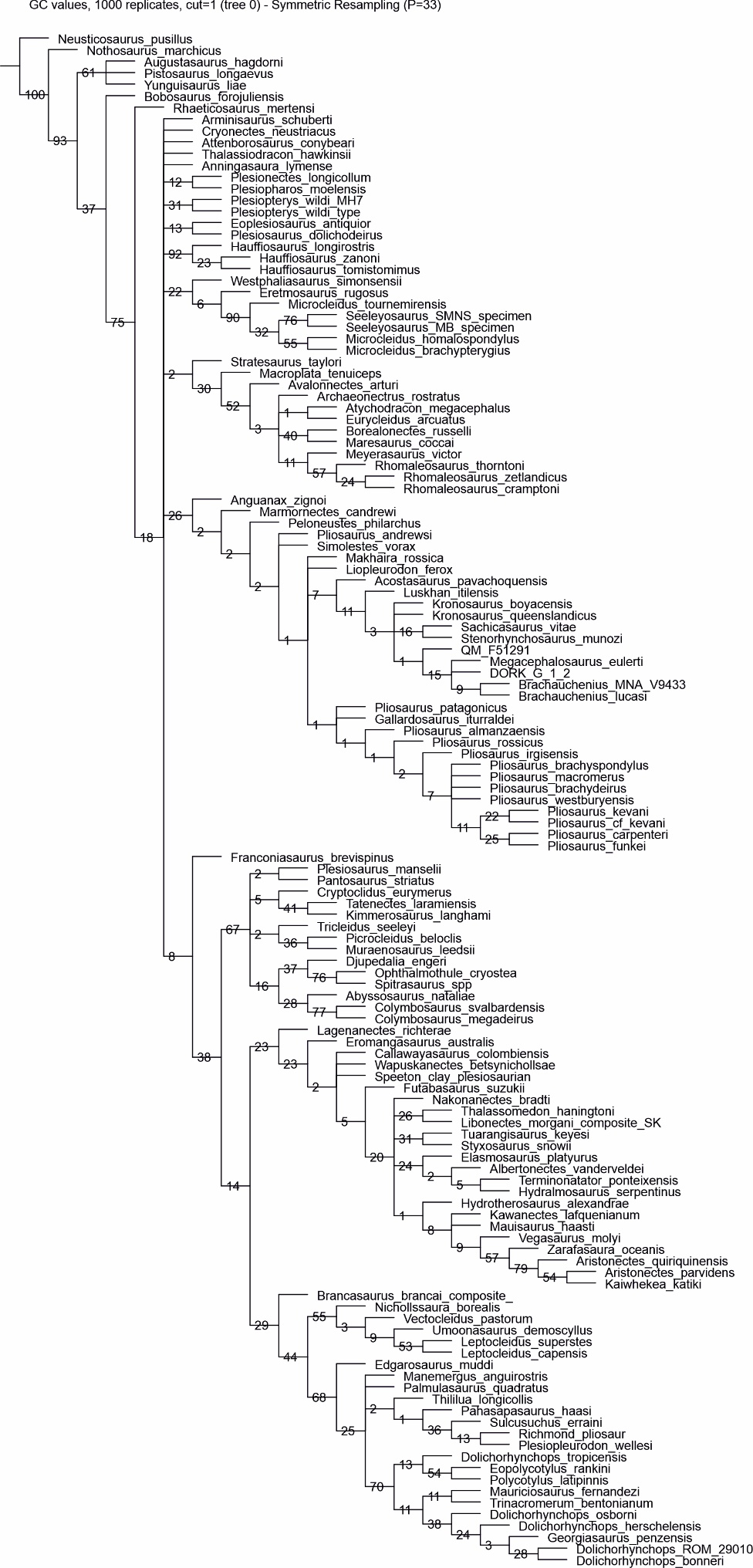

Supplement: Supplemental Information 3 [file peerj-13-19665-s003.docx]
