# Supplementary material for: An unusual early-diverging plesiosauroid from the Lower Jurassic Posidonia Shale of Holzmaden, Germany
Source: PeerJ. 2025 Aug 4;13:e19665. doi: 10.7717/peerj.19665 (PMC12330822; doi:10.7717/peerj.19665)
Supplement: Supplemental Information 4 [file peerj-13-19665-s004.docx]

**Table 1.** Measurements (in mm) of the axial skeleton of *Plesionectes longicollum* (SMNS 51945).

| **Vertebra** | **Centrum length** | **Centrum width** | **Centrum height** | **Neural spine height** | **Neural spine length ventrally** | **Neural spine length dorsally** |
| --- | --- | --- | --- | --- | --- | --- |
| Cervical 1 | 17.90 | 21.00 | - | - | - | - |
| Cervical 2 | 20.40 | - | - | - | - | - |
| Cervical 3 | 20.00 | 24.10 | - | - | - | - |
| Cervical 4 | 22.50 | 25.10 | - | - | - | - |
| Cervical 5 | 21.90 | 26.10 | - | - | - | - |
| Cervical 6 | 23.60 | 28.50 | - | - | - | - |
| Cervical 7 | 26.40 | 30.30 | - | - | - | - |
| Cervical 8 | 24.50 | 34.00 | - | - | - | - |
| Cervical 9 | 27.20 | 33.30 | - | - | - | - |
| Cervical 10 | 26.20 | 34.40 | - | - | - | - |
| Cervical 11 | 25.90 | 32.70 | - | - | - | - |
| Cervical 12 | 28.70 | 34.50 | - | 20.10 | 21.80 | 18.30 |
| Cervical 13 | 30.10 | 24.90 | - | 23.60 | 20.50 | 18.60 |
| Cervical 14 | 27.80 | 35.20 | - | 23.20 | 23.80 | 18.60 |
| Cervical 15 | 29.40 | 35.30 | - | 26.10 | 23.80 | 24.60 |
| Cervical 16 | 31.00 | 26.10 | - | 29.00 | 22.40 | 23.30 |
| Cervical 17 | 30.10 | 36.20 | - | 28.10 | 34.70 | 22.00 |
| Cervical 18 | 32.00 | 36.50 | - | 31.70 | 26.40 | 23.90 |
| Cervical 19 | 33.80 | 37.40 | - | 29.50 | 29.40 | 24.70 |
| Cervical 20 | 32.70 | 33.40 | - | 32.70 | 28.10 | 26.60 |
| Cervical 21 | 34.00 | - | - | 34.30 | 26.90 | 26.20 |
| Cervical 22 | 25.00 | - | - | 33.20 | 28.40 | 26.80 |
| Cervical 23 | 33.40 | - | - | 33.60 | 29.00 | 27.10 |
| Cervical 24 | 35.30 | 43.60 | - | 35.80 | 28.60 | 27.60 |
| Cervical 25 | 39.90 | 40.00 | - | 37.90 | 29.30 | 27.60 |
| Cervical 26 | 34.70 | 41.70 | - | 41.00 | 31.10 | 28.80 |
| Cervical 27 | 36.30 | 43.60 | - | 38.90 | 29.30 | 28.50 |
| Cervical 28 | 39.00 | 44.50 | - | 43.80 | 29.30 | 27.60 |
| Cervical 29 | 35.50 | - | - | 45.00 | 29.50 | 27.40 |
| Cervical 30 | 35.20 | - | - | 46.00 | 29.70 | 28.00 |
| Cervical 31 | 35.60 | - | - | 47.00 | 30.80 | 30.70 |
| Cervical 32 | 36.30 | - | - | 47.70 | 30.60 | 28.20 |
| Cervical 33 | 39.00 | - | - | 49.60 | 31.80 | 28.70 |
| Cervical 34 | 39.20 | - | - | 50.30 | 29.30 | 27.60 |
| Cervical 35 | 37.50 | - | - | 52.90 | 29.40 | 28.50 |
| Cervical 36 | 35.50 | - | - | 51.50 | 28.50 | 29.00 |
| Cervical 37 | 37.70 | - | - | 53.30 | 30.90 | 29.30 |
| Cervical 38 | 37.40 | - | - | 55.70 | 28.80 | 30.40 |
| Cervical 39 | 36.90 | - | - | 55.90 | 28.70 | 28.40 |
| Cervical 40 | 35.90 | - | - | 55.30 | 28.20 | 27.30 |
| Cervical 41 | 33.70 | - | - | 55.30 | 26.80 | 27.90 |
| Pectoral 1 | 37.50 | - | - | 53.00 | 26.80 | 28.90 |
| Pectoral 2 | 33.50 | - | - | 53.70 | 25.40 | 30.30 |
| Pectoral 3 | 38.50 | - | - | 51.10 | 27.80 | 27.30 |
| Pectoral 4 | 35.70 | - | - | 49.60 | 25.50 | 28.20 |
| Dorsal 1 | 37.00 | - | - | - | - | 27.40 |
| Dorsal 2 | 35.40 | - | - | - | - | - |
| Dorsal 3 | 36.70 | - | - | - | - | - |
| Dorsal 4 | 35.10 | - | - | - | - | - |
| Dorsal 5 | 35.80 | - | - | - | - | - |
| Dorsal 6 | 34.00 | - | - | - | - | - |
| Dorsal 7 | - | - | - | - | - | - |
| Dorsal 8 | 35.00 | - | - | - | - | - |
| Dorsal 9 | 37.20 | - | - | - | - | - |
| Dorsal 10 | 38.60 | - | - | - | - | - |
| Dorsal 11 | 33.30 | 42.90 |  | - | 25.80 | 25.00 |
| Dorsal 12 | 37.60 | - | - | - | - | 28.30 |
| Dorsal 13 | 34.80 | - | - | - | - | 29.00 |
| Dorsal 14 | 31.80 | - | - | - | - | 27.80 |
| Dorsal 15 | 34.60 | - | - | 43.20 | 23.50 | 27.60 |
| Dorsal 16 | 29.90 | - | - | 38.40 | 22.50 | 27.10 |
| Dorsal 17 | - | - | - | 41.50 | 22.80 | 24.80 |
| Dorsal 18 | 30.50 | - | - | 40.20 | 22.10 | 22.60 |
| Dorsal 19 | 27.50 | - | - | 34.00 | 20.30 | 22.00 |
| Dorsal 20 | 24.50 | - | - | 35.10 | 20.90 | 22.80 |
| Dorsal 21 | 33.60 | - | - | - | 21.40 | 23.20 |
| Sacral 1 | 28.40 | - | - | 34.40 | 20.60 | 25.00 |
| Sacral 2 | 24.30 | 30.60 | - | 35.40 | 20.40 | 21.70 |
| Caudal 1 | 26.90 | - | 33.70 | 32.00 | 23.60 | 24.20 |
| Caudal 2 | 30.80 | - | 30.70 | 33.50 | 21.00 | 18.50 |
| Caudal 3 | 25.60 | - | 32.30 | 32.20 | 21.70 | 17.80 |
| Caudal 4 | 25.00 | - | 29.70 | 29.40 | - | 19.50 |
| Caudal 5 | 26.70 | - | 27.90 | 27.00 | 18.40 | 17.60 |
| Caudal 6 | 26.70 | - | 30.00 | 29.00 | 19.70 | 19.00 |
| Caudal 7 | 25.80 | - | - | 29.00 | - | 20.80 |
| Caudal 8 | 26.10 | - | - | 30.00 | - | 18.10 |
| Caudal 9 | 26.40 | - | - | 27.30 | 18.80 | 18.50 |
| Caudal 10 | 25.40 | - | - |  | - |  |
| Caudal 11 | 23.40 | - | 33.50 |  | - | 17.40 |
| Caudal 12 | 24.90 | - | 32.10 | 25.10 | - | 14.60 |
| Caudal 13 | 23.00 | - | - | 23.50 | - | 14.30 |
| Caudal 14 | 24.90 | 41.40 | - | 20.90 | - | 15.50 |
| Caudal 15 | 25.00 | 39.80 | - | - | - | - |
| Caudal 16 | 23.60 | - | - | - | - | - |
| Caudal 17 | 22.60 | - | - | - | - | - |
| Caudal 18 | 20.90 | - | - | - | - | - |
| Caudal 19 | 18.90 | - | - | - | - | - |
| Caudal 20 | 20.70 | 32.80 | - | - | - | - |
| Caudal 21 | 19.00 | 30.00 | - | - | - | - |
| Caudal 22 | 19.80 | 31.30 | - | - | - | - |
| Caudal 23 | 20.90 | 31.40 | - | - | - | - |
| Caudal 24 | 20.80 | 29.60 | - | - | - | - |
| Caudal 25 | 20.50 | 28.80 | - | - | - | - |
| Caudal 26 | 18.80 | 30.50 | - | - | - | - |
| Caudal 27 | 18.50 | 22.40 | - | - | - | - |
| Caudal 28 | 18.50 | 22.20 | - | - | - | - |
| Caudal 29 | 16.70 | 21.60 | - | - | - | - |
| Caudal 30 | 16.50 | 21.60 | - | - | - | - |
| Caudal 31 | 17.40 | 18.20 | - | - | - | - |
| Caudal 32 | 12.10 | 20.00 | - | - | - | - |
| Caudal 33 | 10.50 | 17.70 | - | - | - | - |
| Caudal 34 | 23.70 | 18.20 | - | - | - | - |
| Caudal 35 | 13.00 | 15.00 | - | - | - | - |
| Caudal 36 | 11.00 | 14.00 | - | - | - | - |
| Caudal 37 | 9.60 | 14.80 | - | - | - | - |
| Caudal 38 | 6.60 | 13.40 | - | - | - | - |
| Caudal 39 | 8.60 | 12.50 | - | - | - | - |
