# Supplementary material for: An unusual early-diverging plesiosauroid from the Lower Jurassic Posidonia Shale of Holzmaden, Germany
Source: PeerJ. 2025 Aug 4;13:e19665. doi: 10.7717/peerj.19665 (PMC12330822; doi:10.7717/peerj.19665)
Supplement: Supplemental Information 5 [file peerj-13-19665-s005.docx]

**Table 2.** Measurements (in mm) of the limb elements of *Plesionectes longicollum* (SMNS 51945); dist = distally, prox. = proximally.

| **Front limb (left)** | Height proximodistally | Lengths anteroposteriorly |
| --- | --- | --- |
| Humerus | 149.00 | 66.20 dist. \| 46.70 prox. |
| Radius | 39.10 | 34.20 dist. \| 27.30 prox. |
| Ulna | 41.10 | 29.20 dist. \| 35.20 prox. |
| Radiale | 16.90 | 20.00 |
| Ulnare | 20.80 | 21.80 |
| Intermedium | 24.10 | 28.20 |
| Distal carpal I | 11.50 | 11.30 |
| Distal carpal II | 15.50 | 15.70 |
| Distal carpal III | 17.30 | 17.30 |
| Metacarpal I | 14.40 | 6.90 |
| Metacarpal II | 19.00 | 16.00 |
| Metacarpal III | 21.70 | 11.80 |
| Metacarpal IV | 22.90 | 13.50 |
| Metacarpal V | 23.60 | 15.80 |
| Digit II Phalanx 1 | 17.00 | 12.30 |
| Digit II Phalanx 2 | 15.20 | 11.30 |
| Digit II Phalanx 3 | 13.50 | 8.80 |
| Digit II Phalanx 4 | 10.70 | 6.40 |
| Digit II Phalanx 5 | 8.10 | 6.00 |
| Digit II Phalanx 6 | 6.40 | 2.90 |
| Digit III Phalanx 1 | 19.10 | 10.10 |
| Digit III Phalanx 2 | 17.40 | 10.60 |
| Digit III Phalanx 3 | 15.80 | 9.50 |
| Digit III Phalanx 4 | 13.10 | 8.10 |
| Digit III Phalanx 5 | 11.70 | 6.20 |
| Digit III Phalanx 6 | 10.70 | 5.60 |
| Digit III Phalanx 7 | 8.50 | 6.30 |
| Digit III Phalanx 8 | 9.30 | 3.60 |
| Digit IV Phalanx 1 | 22.00 | 12.30 |
| Digit IV Phalanx 2 | 18.90 | 11.30 |
| Digit IV Phalanx 3 | 18.10 | 11.70 |
| Digit IV Phalanx 4 | 15.20 | 8.50 |
| Digit IV Phalanx 5 | 12.70 | 9.20 |
| Digit IV Phalanx 6 | 11.10 | 6.60 |
| Digit IV Phalanx 7 | 9.10 | 5.80 |
| Digit IV Phalanx 8 | 6.30 | 4.10 |
| Digit V Phalanx 1 | 21.30 | 12.70 |
| Digit V Phalanx 2 | 18.80 | 12.40 |
| Digit V Phalanx 3 | 16.20 | 9.70 |
| Digit V Phalanx 4 | 14.80 | 8.40 |
| Digit V Phalanx 5 | 11.20 | 6.20 |
| Digit V Phalanx 6 | 9.30 | 6.10 |
| Digit V Phalanx 7 | 7.30 | 4.30 |
| **Front limb (right)** |  |  |
| Radius | 38.60 | 31.00 dist. \| 40.10 prox. |
| Radiale | 22.00 | 22.40 |
| **Hind limb (left)** |  |  |
| Femur | 170.00 | 86.50 dist. \| 40.00 prox. |
| Tibia | 46.00 | 41.10 dist. \| 48.70 prox. |
| Fibula | 50.00 | 38.00 dist. \| 42.00 prox. |
| Tibiale | 20.80 | 20.90 |
| Fibulare | 26.80 | 28.80 |
| Intermedium | 16.80 | 20.80 |
| Distal tarsal I | - | - |
| Distal tarsal II | 18.60 | 18.30 |
| Distal tarsal III | 19.00 | 20.00 |
| Metatarsal I | - | - |
| Metatarsal II | 23.30 | 18.00 |
| Metatarsal III | 27.90 | 20.00 |
| Metatarsal IV | 25.80 | 21.40 |
| Metatarsal V | 30.00 | 24.00 |
| Digit II Phalanx 1 | 21.30 | 16.00 |
| Digit II Phalanx 2 | 19.60 | 14.50 |
| Digit II Phalanx 3 | 17.40 | 12.80 |
| Digit II Phalanx 4 | 14.10 | 10.40 |
| Digit II Phalanx 5 | 12.10 | 7.70 |
| Digit II Phalanx 6 | 9.50 | 5.00 |
| Digit III Phalanx 1 | 25.70 | 18.40 |
| Digit III Phalanx 2 | 25.80 | 16.60 |
| Digit III Phalanx 3 | 20.00 | 14.60 |
| Digit III Phalanx 4 | 18.60 | 13.70 |
| Digit III Phalanx 5 | 16.00 | 11.60 |
| Digit III Phalanx 6 | 13.00 | 9.10 |
| Digit III Phalanx 7 | 11.40 | 8.00 |
| Digit III Phalanx 8 | 9.70 | 6.00 |
| Digit III Phalanx 9 | 6.60 | 5.30 |
| Digit III Phalanx 10 | 2.40 | 3.50 |
| Digit IV Phalanx 1 | 28.40 | 18.60 |
| Digit IV Phalanx 2 | 25.50 | 17.70 |
| Digit IV Phalanx 3 | 21.30 | 15.30 |
| Digit IV Phalanx 4 | 18.70 | 13.10 |
| Digit IV Phalanx 5 | 15.20 | 11.20 |
| Digit IV Phalanx 6 | 13.20 | 10.00 |
| Digit IV Phalanx 7 | 11.60 | 9.80 |
| Digit IV Phalanx 8 | 9.70 | 7.20 |
| Digit IV Phalanx 9 | 5.70 | 3.40 |
| Digit V Phalanx 1 | 27.00 | 19.00 |
| Digit V Phalanx 2 | 22.90 | 15.00 |
| Digit V Phalanx 3 | 20.20 | 13.50 |
| Digit V Phalanx 4 | 18.10 | 10.80 |
| Digit V Phalanx 5 | 14.00 | 9.30 |
| Digit V Phalanx 6 | 12.80 | 8.00 |
| Digit V Phalanx 7 | 9.40 | 7.40 |
| Digit V Phalanx 8 | 4.40 | 3.30 |
| **Hind limb (right)** |  |  |
| Femur | 170.00 | 78.00 dist. \| 41.90 prox. |
| Tibia | 45.50 | - |
| Fibula | 49.60 | 36.00 dist. \| 40.80 prox. |
