# Supplementary material for: An unusual early-diverging plesiosauroid from the Lower Jurassic Posidonia Shale of Holzmaden, Germany
Source: PeerJ. 2025 Aug 4;13:e19665. doi: 10.7717/peerj.19665 (PMC12330822; doi:10.7717/peerj.19665)
Supplement: Supplemental Information 6 [file peerj-13-19665-s006.docx]

**Table 3.** Measurements (in mm) of the girdle elements of *Plesionectes longicollum* (SMNS 51945).

| \| **Interclavicle** \|  \| \| --- \| --- \| \| Length anteroposterioly \| 80.80 \| \| Width anteriorly \| 71.70 \| \|  \|  \| \| **Scapula (left)** \|  \| \| Length of ventral plate \| 93.40 \| \| Height of ventral plate at posterior end \| 25.80 \| \| Height of anterior end of scapula \| 33.60 \| \| Dorsal process. length along anterior edge \| 77.70 \| \| Dorsal process. length at ventral base \| 63.60 \| \| Dorsal process. length at dorsal end \| 28.90 \| \| Dorsal process. height posteriorly \| 48.70 \| \|  \|  \| \| **Pubis (right)** \|  \| \| Largest width \| 104.50 \| \|  \|  \| \| **Ischium (right)** \|  \| \| Length laterally \| 48.20 \| \| Width \| 91.00 \| \|  \|  \| \| **Ischium (left)** \|  \| \| Length medially \| 94.50 \| \|  \|  \| \| **Ilium (left)** \|  \| \| Height dorsoventrally \| 90.70 \| \| Length anteroposteriorly at dorsal end \| 42.60 \| \| Length anteroposteriorly at ventral end \| 33.20 \| \|  \|  \| \| **Ilium (right)** \|  \| \| Length anteroposteriorly at dorsal end \| 47.50 \| |
| --- | --- | --- | --- | --- | --- | --- | --- | --- | --- | --- | --- | --- | --- | --- | --- | --- | --- | --- | --- | --- | --- | --- | --- | --- | --- | --- | --- | --- | --- | --- | --- | --- | --- | --- | --- | --- | --- | --- | --- | --- | --- | --- | --- | --- | --- | --- | --- | --- | --- | --- | --- | --- | --- | --- | --- | --- | --- | --- | --- | --- |
